# Differential diagnosis of benign and malignant vertebral compression fractures using conventional and advanced MRI techniques

**DOI:** 10.1259/bjro.20180033

**Published:** 2019-05-12

**Authors:** Valeria Romeo, Lorenzo Ugga, Arnaldo Stanzione, Sirio Cocozza, Renato Cuocolo, Arturo Brunetti

**Affiliations:** Deparment of Advanced Biomedical Sciences, University of Naples Federico II, Via S. Pansini, Naples, Italy

## Abstract

Atraumatic vertebral compression fractures (VCFs) are commonly encountered in clinical practice and often represent a diagnostic challenge. MRI plays a major role in the differential diagnosis of benign and malignant VCFs, due to its high contrast resolution and the possibility to obtain quantitative and functional data with the employment of advanced sequences. Computer-aided diagnosis systems are also applied on MRI images for this purpose, showing promising results. In this setting, aim of this pictorial review is to elucidate the role of MRI in the differential diagnosis of VCFs with a specific focus on advanced and post-processing imaging techniques.

## introduction

Patients with atraumatic vertebral compression fractures (VCFs) may often represent a diagnostic conundrum, with specific regard to the elderly population. Indeed, VCFs are known to be caused by a broad spectrum of conditions, both benign and malignant, thus rendering hard the differential diagnosis. Osteoporosis and metastatic disease can be considered the most common benign and malignant causes of VCFs, respectively. While patient clinical history could help raising suspicion for a particular disorder, the characterization of VCFs is usually referred to diagnostic imaging. Among the available imaging modalities, MRI is increasingly gaining relevance in this field due to its high contrast resolution and the possibility to obtain both morphologic and functional information by means of advanced sequences as well as post-processing imaging techniques.

Aim of this review is to elucidate the role of MRI in the differential diagnosis of VCFs with a specific focus on advanced and post-processing imaging techniques.

## conventional MRI

### Morphologic features, signal intensity and enhancement patterns

**Figure 1.**
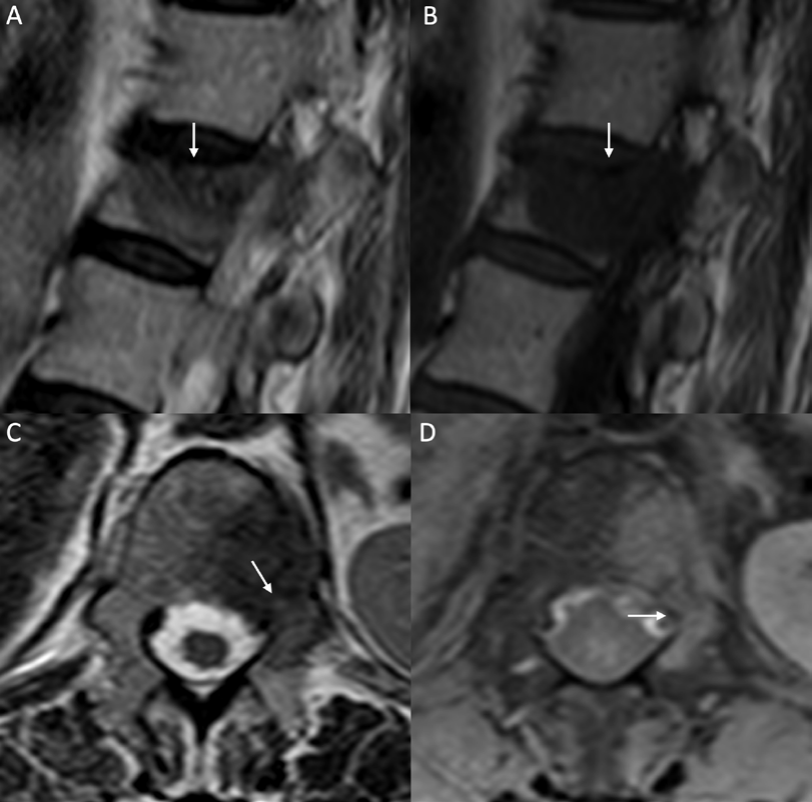
Malignant VCF. Sagittal *T*_2_W (A), sagittal *T*_1_W (B), axial *T*_2_W (C) and axial post-contrast *T*_1_W with fat saturation (D) images. Height loss of D12 with a large area of abnormal signal intensity (arrow in A), hypointense on *T*_1_W-image (B), also involving the left pedicle (arrow in C) and showing abnormal enhancement on post-contrast images (white arrow in D), consistent with a metastasis from breast cancer. VCF, vertebral compression fracture.

**Figure 2.**
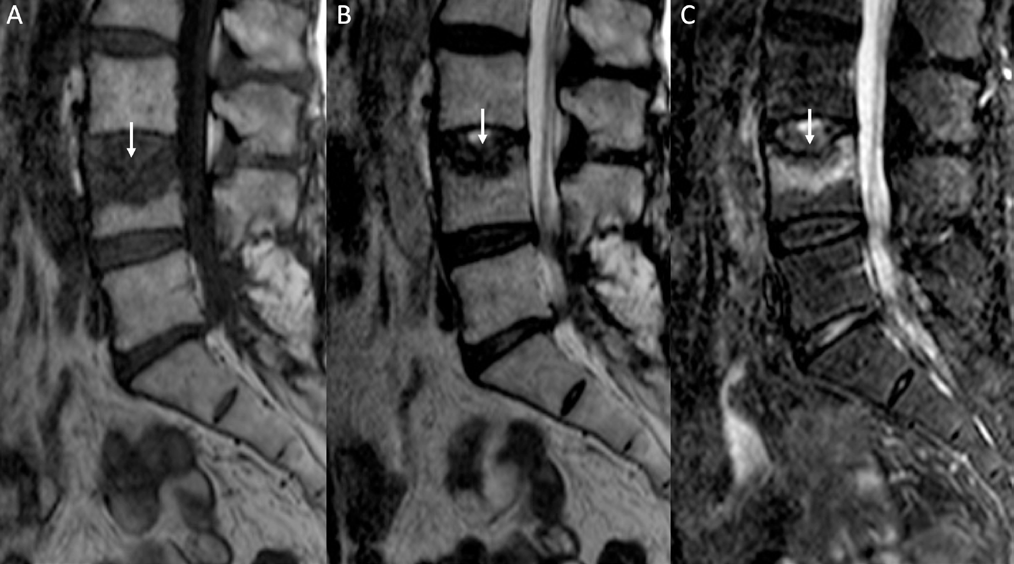
Benign VCF. Sagittal *T*_1_W (A), *T*_2_W (B) and STIR-W (C) images. Height loss of L4 showing area of low signal on *T*_1_W images (white arrow in A), a smaller hypointense area on *T*_2_W images (white arrow in B) and increased signal intensity on STIR images consistent with intramedullary edema (white arrow in C). STIR, short tau inversion recovery; VCF, vertebral compression fracture.

**Figure 3.**
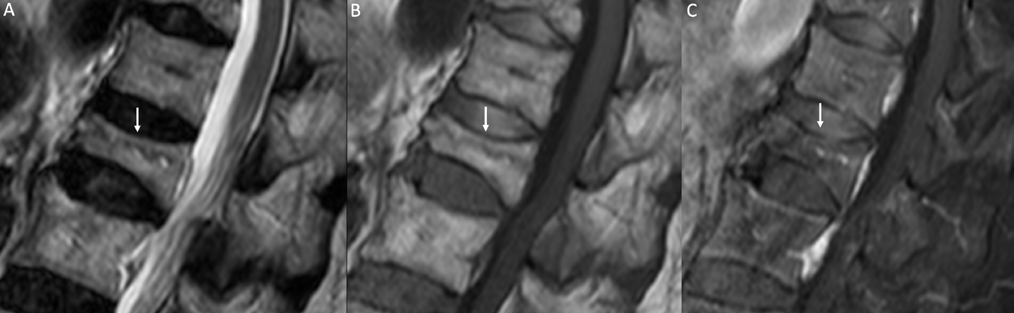
Benign VCF. Sagittal *T*_2_W (A), *T*_1_W (B), and post-contrast *T*_1_W with fat saturation (C) images. Height loss of D11 with a horizontal line within the vertebral body consistent with linear fracture (white arrows in A and B). After contrast administration, no abnormal enhancement is detectable (C). VCF, vertebral compression fracture.

**Figure 4.**
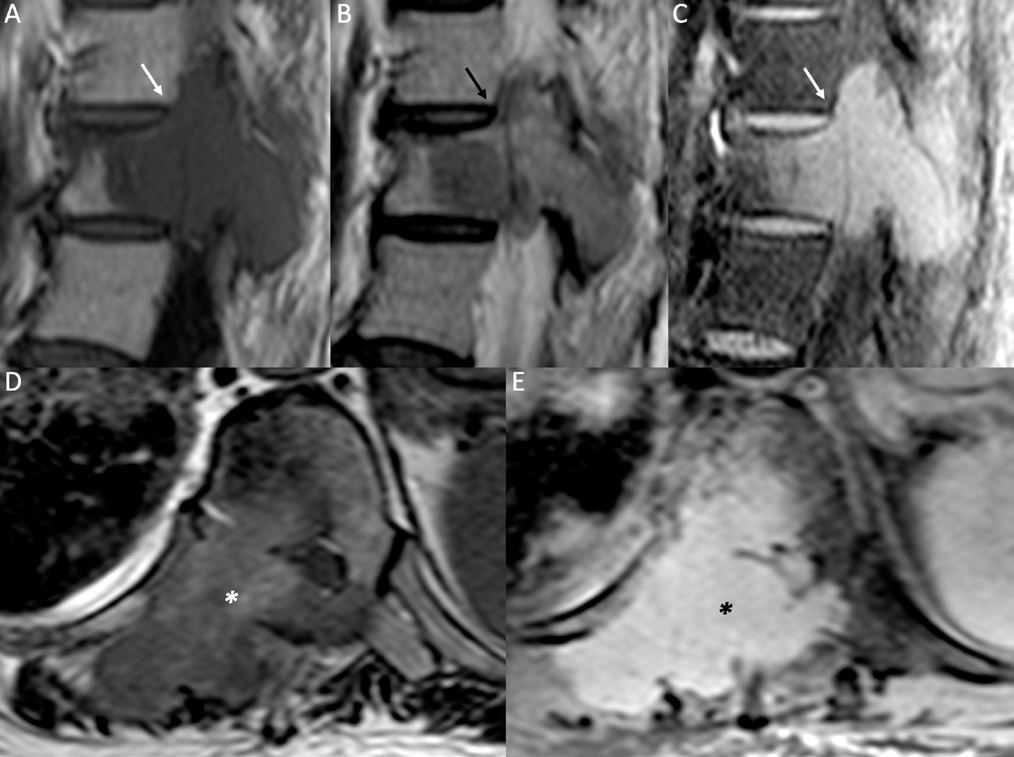
Malignant VCF. Sagittal *T*_1_W (A), *T*_2_W (B) and post-contrast *T*_1_W with fatsaturation (C) images, axial *T*_2_W (D) and post-contrast *T*_1_W with fat saturation (E) images. A large area of abnormal signal intensity is detectable at the posterior vertebral body and right pedicle of D12, hypointense on *T*_1_W and *T*_2_W images (white arrow in A and black arrow in B), showing heterogeneous enhancement on post-contrast images (white arrow in C). A soft tissue extending through the right paravertebral space is also present, showing low signal intensity on *T*_2_W images (white asterisk in D) and irregular enhancement on post-contrast images (black asterisk in E). This finding was proved to be a metastasis from thymic carcinoma. VCF, vertebral compression fracture.

**Figure 5.**
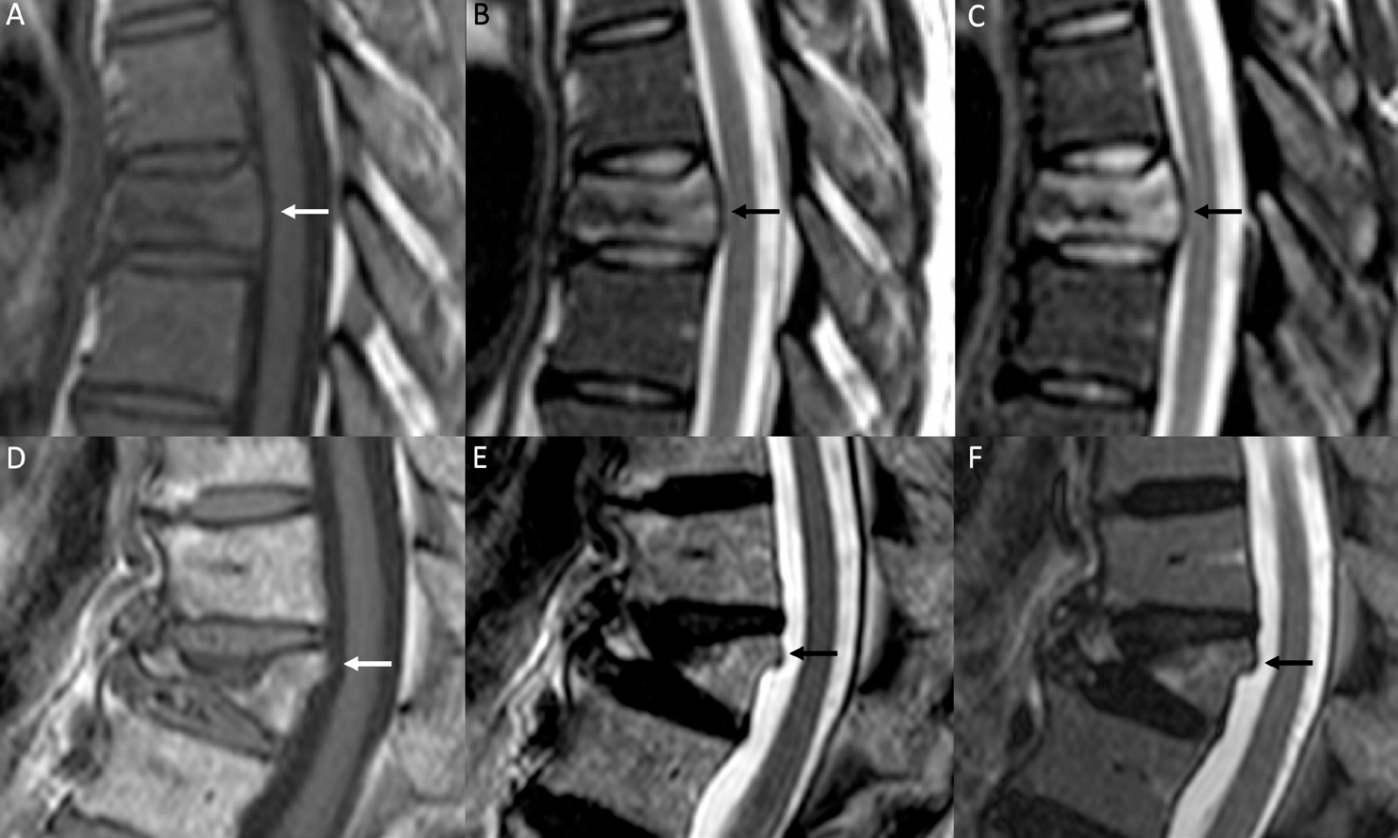
Posterior wall involvement in VCFs. A smoothly blunt protrusion of the posterior wall of the vertebral body is shown in a malignant VCF in a patient with non-Hodgkin lymphoma (A, B and C, *T*_1_W, *T*_2_W and STIR images respectively). On the other hand, a retropulsion of the superoposterior body corner is present in a benign VCF (D, E and F, *T*_1_W, *T*_2_W and STIR images respectively). STIR, short tau inversion recovery; VCF, vertebral compression fracture.

Conventional MRI features may aid in the differential diagnosis of benign and malignant VCFs, even if an overlap between the two entities may often occur. A typical MRI feature suggestive of malignancy is the presence of abnormal bone marrow signal intensity involving the pedicles or other posterior vertebral elements.^1^ Malignant VCFs often have total replacement of the high *T*_1_ bone marrow signal intensity resulting in diffuse homogeneous low signal and showing heterogeneous enhancement after contrast administration (Figure 1). However, osteoporotic fractures may commonly show similar signal changes in the acute phase due to marrow edema extension (Figure 2), while a malignant vertebral fracture may have preserved pedicles signal intensity if there is no tumor infiltration at that level. The presence of a linear horizontal band of low *T*_1_ and *T*_2_ signal intensity, parallel and adjacent to the endplate, is a specific sign of benignity, representing the fracture line or trabeculae compaction. Fluid sign, a strong indicator of benign VCF, refers to a cleft of marked *T*_2_ hyperintensity within the collapsed vertebra due to a fluid collection in an osteonecrosis area. After gadolinium injection, benign fractures tend to have enhancement similar to adjacent normal vertebrae (Figure 3).^2^ The presence of epidural or paravertebral soft-tissue abnormalities is also suspect for a pathologic VCF, representing direct neoplastic extension from the vertebrae into the adjacent spaces which tends to be mass-like (Figure 4). Nevertheless, even this feature may be not specific in case of paravertebral or epidural hemorrhage with associated edema in benign VCFs. A smoothly blunt protrusion of the posterior wall of the vertebral body implies a malignant fracture, due to the bulging mass into the anterior epidural space; on the other hand, if a retropulsion of bone fragments from vertebral body corners is present, the fracture is more likely to be benign (Figure 5).

### Chemical shift imaging

Chemical shift imaging is based on the different behavior in resonance frequency of hydrogen protons contained in water and lipids, providing therefore information about their concentration. Since malignant infiltrative processes tend to replace the fatty marrow components completely, the detection of fat content within bone marrow through a drop in signal intensity greater than 35% in out of phase images was proved to be useful to differentiate benign from malignant VCFs, although with lower diagnostic accuracy compared to other imaging techniques.^3^ However, the presence of sclerosis, or lipid-enriched lesions (*i.e.* multiple myeloma) could lead to false-positive and negative results, respectively. Six-echo Dixon MRI sequences have also been applied showing a good diagnostic accuracy of fat fraction and fat fraction ratio in the discrimination of osteoporotic and malignant VCFs, with area under the curve values of 0.98 and 0.95, respectively.^4^ MRI findings of benign and malignant VCFs are summarized in Table 1.

**Table 1.** Summary of morphological and chemical-shift MRI findings of benign and malignant VCFs

**Imaging technique**	**Benign VCFs findings**	**Malignant VCFs findings**
***T*_1_ weighted and *T*_2_-weighted**	Regular bone marrow signal intensity Fluid sign Low *T*_1_ and *T*_2_ signal linear horizontal band Retropulsion of bone fragments from vertebral body corners	Diffuse and homogeneous hypointensity often involving pedicles or posterior elements Epidural or paravertebral soft tissue abnormalities Convexity of the posterior wall of the vertebral body
**Post-contrast *T*_1_ weighted**	Enhancement pattern similar to adjacent vertebrae	Heterogeneous and increased enhancement
**Chemical shift**	Drop of signal on out-phase images Normal fat fraction ratio on Dixon images	Decreased or absent drop of signal on out-phase images Decreased fat fraction ratio on Dixon images

Note: VCFs = vertebral compression fractures.

## advanced MRI techniques

### Diffusion weighted imaging

Water molecules diffusion is usually increased in benign fractures due to bone marrow edema, while malignant fractures show diffusion restriction related to the high cellularity.

Besides qualitative evaluation, diffusion-weighted imaging (DWI) can also be quantitatively assessed by calculating the apparent diffusion coefficient (ADC) value. Several MRI sequences have been tested but, unfortunately, all published studies show a remarkable overlap between benign and malignant ADC values, mostly due to the presence of intravertebral hematoma in benign lesions. However, DWI may provide beneficial information in combination with conventional imaging improving sensitivity, specificity and accuracy.^5^ In this setting, ADC maps calculated with a combination of low to intermediate *b* values (*b* = 100, 250, and 400 s/mm2) provided the best diagnostic performance to differentiate acute benign and malignant VCFs with a cutoff ADC < 1.7×10–3 mm^2^/s.^6^

### Dynamic contrast enhanced imaging

Dynamic contrast-enhanced MRI is an advanced imaging technique assessing tissue perfusion and microvasculature through the extraction of semi-quantitative and quantitative parameters that reflect kinetics and hemodynamics vasculature features.^7^ Among semiquantitative parameters peak enhancement, steepest slope and slope values were found significantly different between pathologic and osteoporotic VCFs, while conflicting results are reported regarding the role of time intensity curves pattern to discriminate the two entities.^8,9^

**Figure 6.**
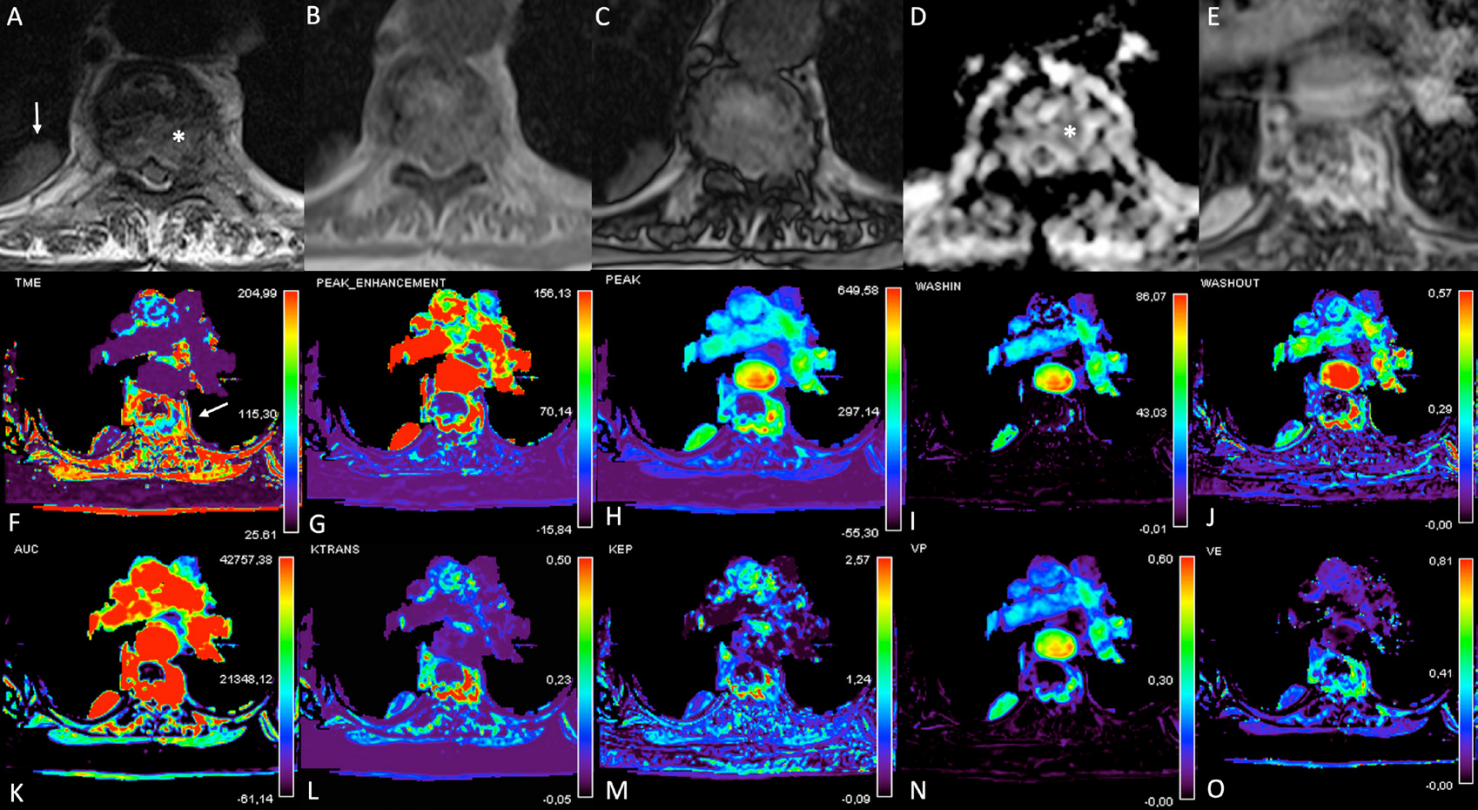
MRI multiparametric evaluation of a malignant VCF. Axial *T*_2_W (A), *T*_1_W “in phase” (B), *T*_1_W “out of phase” (C), ADC map (D) and dynamic post-contrast (E) images; time to maximum enhancement (F), peak enhancement (G), peak (H), wash-in (I), wash-out (J), AUC (K), Ktrans (L), Kep (M), plasma volume (N), Ve (O) perfusion maps. A vertebral metastasis from renal carcinoma is detected as a heterogeneous bone marrow signal abnormality involving D5 vertebral body and left pedicle with epidural extension on *T*_2_ weighted images (asterisk in A), showing no signal drop on “out of phase” (C) as compared to “in-phase” (B) images and with restricted diffusivity on ADC map (asterisk in D). Dynamic sequence (E) and perfusion maps of semi-quantitative (F-K) and quantitative (L–O) parameters (white arrow in F) are reported. The lesion showed increased perfusion values with early wash-in and late wash-out, suggestive of the malignant nature. An incidental pneumonia is also appreciable in the right lung (white arrow in A). ADC, apparent diffusion coefficient; AUC, area under the curve; VCF, vertebralcompression fracture.

Differently from semi-quantitative measurements, quantitative parameters can describe tissue hemodynamic features on a biological basis, being able to depict alterations of the microvascular structure related to tumor neoangiogenesis that could lead to increased permeability and different contrast agent transfer between vascular and extravascular spaces.^7^ Quantitative parameters resulted significantly different between malignant and benign VCFs, even if the available studies differ in terms of the kinetic model applied and perfusion parameters extracted. In detail, Vp and Ktrans resulted significantly higher in malignant as compared to benign VCFs, while interstitial volume and the total extracellular volume were significantly higher in benign as compared to malignant VCFs.^10,11^ A multiparametric evaluation of VCFs is illustrated in Figure 6.

### Magnetic resonance spectroscopy

Magnetic resonance spectroscopy is an *in vivo* non-invasive imaging technique that allows a quantitative assessment of the biochemical structure within a specific tissue. Although showing early promising results, being able to determine vertebral lipid content in healthy controls, this technique shows a high between-subject heterogeneity, influenced by several factors including local environment and surface coil.^12^ For these reasons, also considering the availability of more feasible and accurate diagnostic tools, magnetic resonance spectroscopy is not routinely performed in the evaluation of patients with VCFs.

### PET/MRI

2-(^18^F)flu-2-deoxy-d-glucose PET/CT has been widely used for detecting vertebral metastases in oncologic patients. Although malignant fractures are expected to demonstrate different accumulation patterns and a higher 2-(^18^F)flu-2-deoxy-d-glucose uptake as compared to benign fractures with a reported standard uptake value (SUV) threshold ranging from 3 to 4.5, high SUV values have been found in not tumor-related VCFs during the acute phase; a 3 months follow-up is recommended in such cases to demonstrate a normalization of SUV values.^2,13^

At present, PET/CT is considered as an adjunctive imaging technique when MRI findings are not conclusive, showing higher sensitivity but lower sensitivity. In this setting, a combined PET/MRI approach could have a role to further improve the diagnostic accuracy in discriminate benign from malignant VCFs.

## Post-processing imaging techniques

### Texture analysis

**Figure 7.**
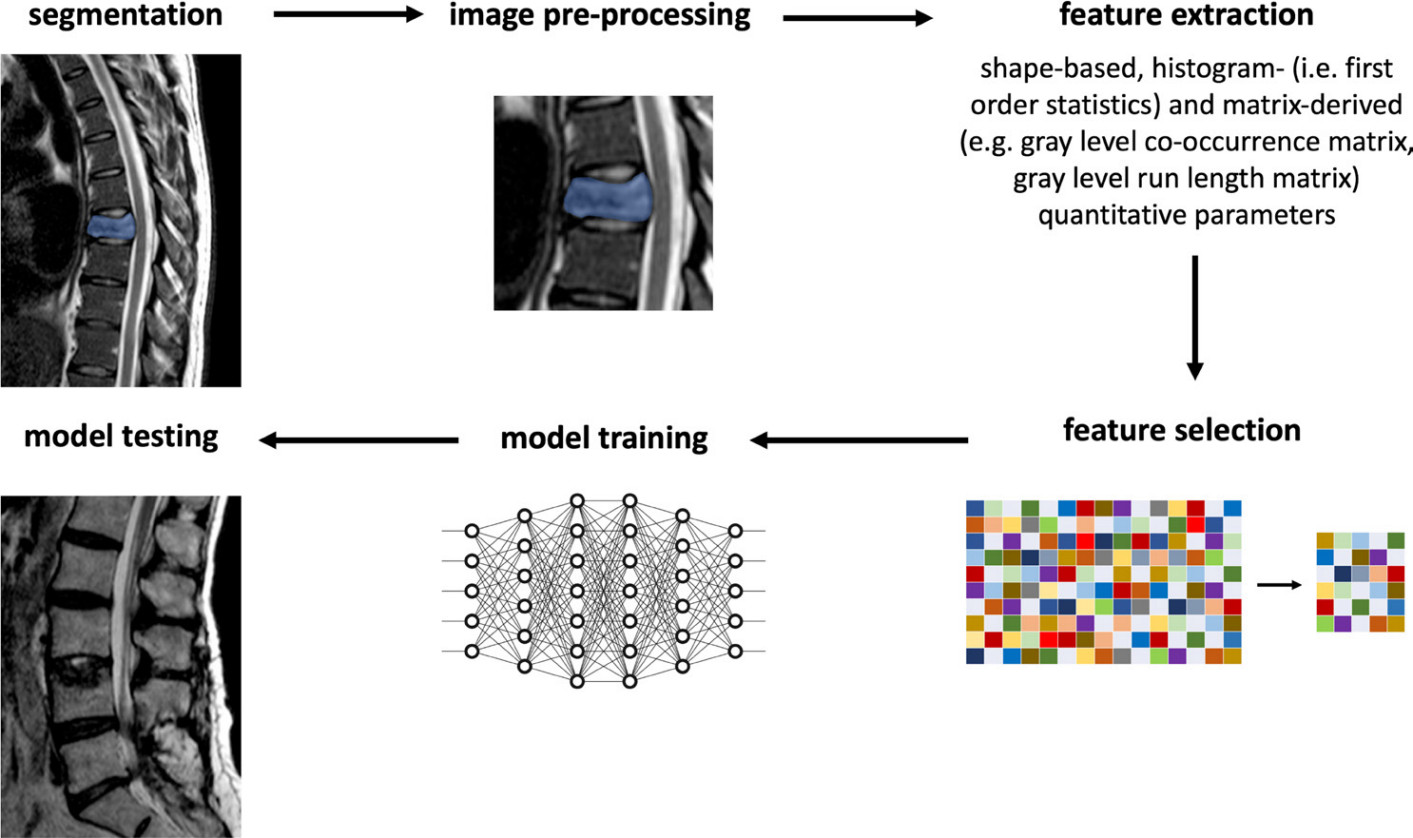
Texture analysis and machine learning workflow. Post-processing of MRI images is illustrated, including vertebral body segmentation, texture feature extraction/selection, and machine learning analysis.

Texture analysis (TA) quantifies the heterogeneity of an image by analyzing the signal intensity through gray level value distribution, allowing for the extraction of a great number of parameters. These can be analyzed through univariate and multivariate statistical tests or can be used in combination with machine learning (ML) classifiers, enabling computers to learn how to classify data without prior explicit programming. ML and TA have been proven useful in the differential diagnosis of benign and malignant VCFs. In this setting, the employment of statistical measures of gray levels, texture features, shape factors extracted from *T*_1_ weighted images and a commonly used ML classifier showed an area under the characteristic curve (AUC) of 0.92 in distinguishing benign from malignant VCFs.^14^ TA and ML workflow is reported in Figure 7.

### Fractal imaging

Fractal dimension provides a way to quantify the shape complexity of an object that represents self-similarity. Several attempts have been made in order to apply fractal analysis to the evaluation of different human body organs showing fractal geometry, including bone trabeculation. One of the major attractions of fractal features is that they are less influenced by randomness and roughness of medical images and thus suitable for the evaluation of MR images in which a certain degree of noise is often encountered. Similar to TA, fractal features extracted from *T*_1_ weighted MR images were employed using a ML classifier and found useful in the classification of benign and malignant VCFs with an AUC value up to 0.95.^15^

**Figure 8.**
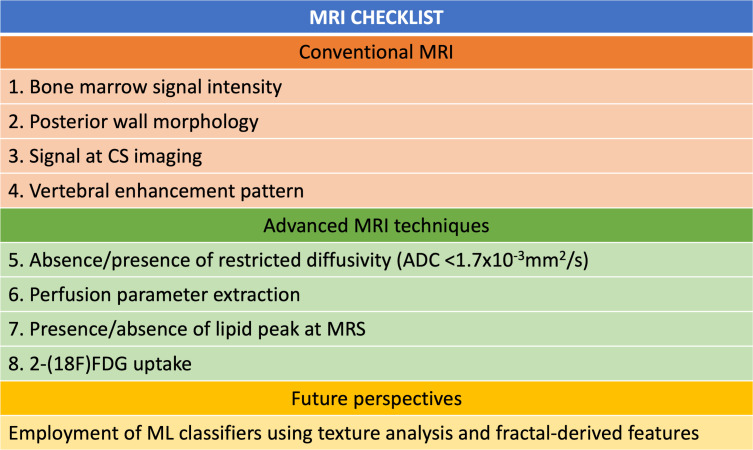
MRI evaluation checklist. Conventional and advanced features to be assessed in order to characterize VCFs. ADC, apparent diffusion coefficient; 2-(^18^F)FDG, 2-(^18^F)flu-2-deoxy-d-glucose;MRS,MR spectroscopy; VCF, vertebral compression fracture.

A checklist summarizing conventional and advanced MRI findings to be assessed in order to characterize VCFs is shown in Figure 8.

## Conclusion

Conventional MRI plays an important role in the differential diagnosis of benign and malignant VCFs due to its superb contrast resolution. Furthermore, the possibility to employ advanced MRI sequences as well as to combine MRI findings with metabolic imaging may enable the depiction of biological processes underlying VCFs. Finally, promising results concerning the diagnostic accuracy of post-processing imaging techniques in discriminating benign and malignant VCFs could lead to the development of dedicated computer-aided diagnosis systems.
